# Nanopore-Based Detection of Viral RNA Modifications

**DOI:** 10.1128/mbio.03702-21

**Published:** 2022-05-17

**Authors:** Jonathan S. Abebe, Ruth Verstraten, Daniel P. Depledge

**Affiliations:** a Department of Microbiology, New York University School of Medicine, New York, New York, USA; b Institute of Virology, Hannover Medical School, Hannover, Germany; c German Center for Infection Research (DZIF), partner site Hannover-Braunschweig, Hannover, Germany; Ohio State University

**Keywords:** RNA modifications, m^6^A, nanopore sequencing, virus

## Abstract

The chemical modification of ribonucleotides plays an integral role in the biology of diverse viruses and their eukaryotic host cells. Mapping the precise identity, location, and abundance of modified ribonucleotides remains a key goal of many studies aimed at characterizing the function and importance of a given modification. While mapping of specific RNA modifications through short-read sequencing approaches has powered a wealth of new discoveries in the past decade, this approach is limited by inherent biases and an absence of linkage information. Moreover, in viral contexts, the challenge is increased due to the compact nature of viral genomes giving rise to many overlapping transcript isoforms that cannot be adequately resolved using short-read sequencing approaches. The recent emergence of nanopore sequencing, specifically the ability to directly sequence native RNAs from virus-infected host cells, provides not just a new methodology for mapping modified ribonucleotides but also a new conceptual framework for what can be derived from the resulting sequencing data. In this minireview, we provide a detailed overview of how nanopore direct RNA sequencing works, the computational approaches applied to identify modified ribonucleotides, and the core concepts underlying both. We further highlight recent studies that have applied this approach to interrogating viral biology and finish by discussing key experimental considerations and how we predict that these methodologies will continue to evolve.

## INTRODUCTION

Since the discovery of pseudouridine (ψ) in 1957 (1), over 170 chemically modified ribonucleotides have been identified (2). Despite this, our knowledge of the function and modulation of modified ribonucleotides is generally sparse, with a few important exceptions. Within the eukaryotic cell, all major RNA species harbor modified ribonucleotides (3–9), with rRNAs and tRNAs among the most heavily modified species (reviewed in references 10 and 11). To date, the best characterized and most widely studied RNA modification is *N*^6^-methyladenosine (m^6^A), first discovered in 1975 (12, 13). This modification is found in mRNA (12), long intergenic noncoding RNAs (lincRNAs) (14), primary microRNAs (pri-miRNAs) (7), and rRNAs (15, 16). For mRNAs, miRNAs, and lincRNAs, m^6^A is installed cotranscriptionally in the nucleus by a methyltransferase complex minimally comprising METTL3 and METTL14 and enhanced by WT1 associated protein (WTAP) and a range of RNA polymerase II accessory proteins (17–19). m^6^A is recognized and bound by multiple YTH family members (YTHDF1-3, YTHDC1-2) along with a variety of hnRNPs and multiple members of the IGF2BP family (20–23). m^6^A is considered a reversible modification and may be removed by the demethylases ALKBH5 and FTO (24–26). While installation of m^6^A generally occurs in DRACH (D = A/G/U; R = G/A; H = A/C/U) sequence contexts, most DRACH sequences are not modified (27). m^6^A has been implicated in regulating numerous diverse cellular processes involved in RNA maturation and function, including splicing, polyadenylation, export, translation, and decay (24, 28–32). Beyond m^6^A, other important mRNA modifications include pseudouridine (ψ), *N*^6^,2′-*O*-dimethyladenosine (m^6^Am) (26, 33–36), 5-methylcytidine (m^5^C) (37, 38), 1-methyladenosine (m^1^A) (39–41), 7-methylguanosine (m^7^G) (42–44), and 2′-*O*-methylation (Nm) (45–47). For an extended overview of these, we direct the reader to the MODOMICS database resource (http://genesilico.pl/modomics) (2).

Unsurprisingly, the influence of RNA modifications on viral biology is complex, and many viruses have evolved exquisite mechanisms for interacting with RNA modification pathways. For viruses which replicate in the nucleus, m^6^A installed on host and viral mRNAs has been shown to have both proviral and antiviral effects (48). These include regulating viral gene expression in the context of HIV-1 (49–51), impairing nuclear processing and export in simian virus 40 (SV40) (52, 53), modulating the splicing ability of adenoviral transcripts (54), preventing m^6^A installation on beta interferon-encoding mRNAs to inhibit type 1 interferon responses (55, 56), and regulating the life cycle of Kaposi’s sarcoma-associated herpesvirus (KSHV) (57–60). The installation of m^6^A on the RNAs of viruses which replicate in the cytoplasm similarly confers proviral or antiviral effects such as interfering with replication of severe acute respiratory syndrome coronavirus-2 (SARS-CoV-2) (61, 62) and enterovirus 71 (63). Other RNA modifications, including pseudouridine, m^5^C, Nm, m^6^Am, *N*^1^-methylguanosine (m^1^G), and *N*^4^-acetylcytidine (ac^4^C), have all been shown to be installed on viral mRNAs (reviewed in references 64 and 65), while modifications such as m^5^C, m^1^A, Nm, m^7^G, m^1^G, and m^6^Am have variously been identified on the genomes of several RNA viruses (66). These modifications all play fundamental roles in viral life cycles. As with the analysis of eukaryotic cells, the location and abundance of modified ribonucleotides have typically been mapped using short-read sequencing and/or mass spectrometry approaches. For one of the most comprehensive overviews to date on the roles of RNA modifications in viral biology, we recommend a recent review by Baquero-Perez et al. (65).

While several techniques enable the identification of RNA modifications on individual RNA isoforms, many of these are low throughput and thus useful only in certain contexts (67). This changed in 2012, when the first high-throughput sequencing approaches for transcriptome-wide mapping of m^6^A were published (27, 68). Termed meRIP-Seq (methylated RNA immunoprecipitation sequencing) or m^6^A-Seq (m^6^A sequencing), these techniques combine immunoprecipitation with antibodies against m^6^A and subsequent short-read (Illumina) sequencing to enrich for RNA fragments containing m^6^A, prepared in parallel with either a nonspecific or no immunoprecipitation control. Once sequenced, reads from m^6^A-containing RNA fragments are enriched over the background control at m^6^A sites and provide a resolution range of 50 to 200 nucleotides (nt). These studies were the first to determine that m^6^A modifications are both abundant and enriched within long internal exons and in 3′ untranslated regions (UTRs). This was later followed by newer antibody-dependent protocols such as miCLIP-Seq (m^6^A individual-nucleotide-resolution cross-linking and immunoprecipitation sequencing) (3), which was used to map m^6^A sites at nucleotide-level resolution. Despite the great success of these protocols, it should be noted that antibody-based approaches suffer from artifacts associated with off-target capture (69). The development of antibody-independent methods such as DART-Seq (deamination adjacent to RNA modification target sequencing) (70), Sequencing of RNA digested via m^6^A sensitive RNase (MAZTER-Seq) (71), m^6^A-label-seq (a metabolic labeling method to detect mRNA m^6^A transcriptome-wide at base resolution) (72), m^6^A-SEAL (an antibody-free, FTO-assisted chemical labeling method) (73), and m^6^A-SAC-seq (m^6^A-selective allyl chemical labeling and sequencing) (74) provide a useful set of orthologous m^6^A-specific methodologies for mapping the locations at which m^6^A is installed. While impressive, these techniques remain constrained by the biases and nonbiological variation associated with short-read sequencing approaches (69, 75), as well as the loss of linkage that comes from fragmenting individual RNA molecules. Moreover, in a viral context, short-read sequencing is generally not suitable for disentangling the complex overlapping RNA isoforms that are characteristic of most viral species (76). While this does not preclude the use of the above discussed approaches to interrogate viral biology, it does limit the conclusions that can be made. With this in mind, we define the ultimate aim of RNA modification detection approaches as follows: to detect, at single-level nucleotide resolution, the presence and identity of all modified ribonucleotides installed on a single RNA. By nature, short-read sequencing approaches cannot fulfill this goal. Indeed, the optimal way to accomplish this would require direct full-length sequencing of RNAs in such a way that the position and identity of any modified ribonucleotides with a given RNA can be discerned.

## NANOPORE DIRECT RNA SEQUENCING

**FIG 1:**
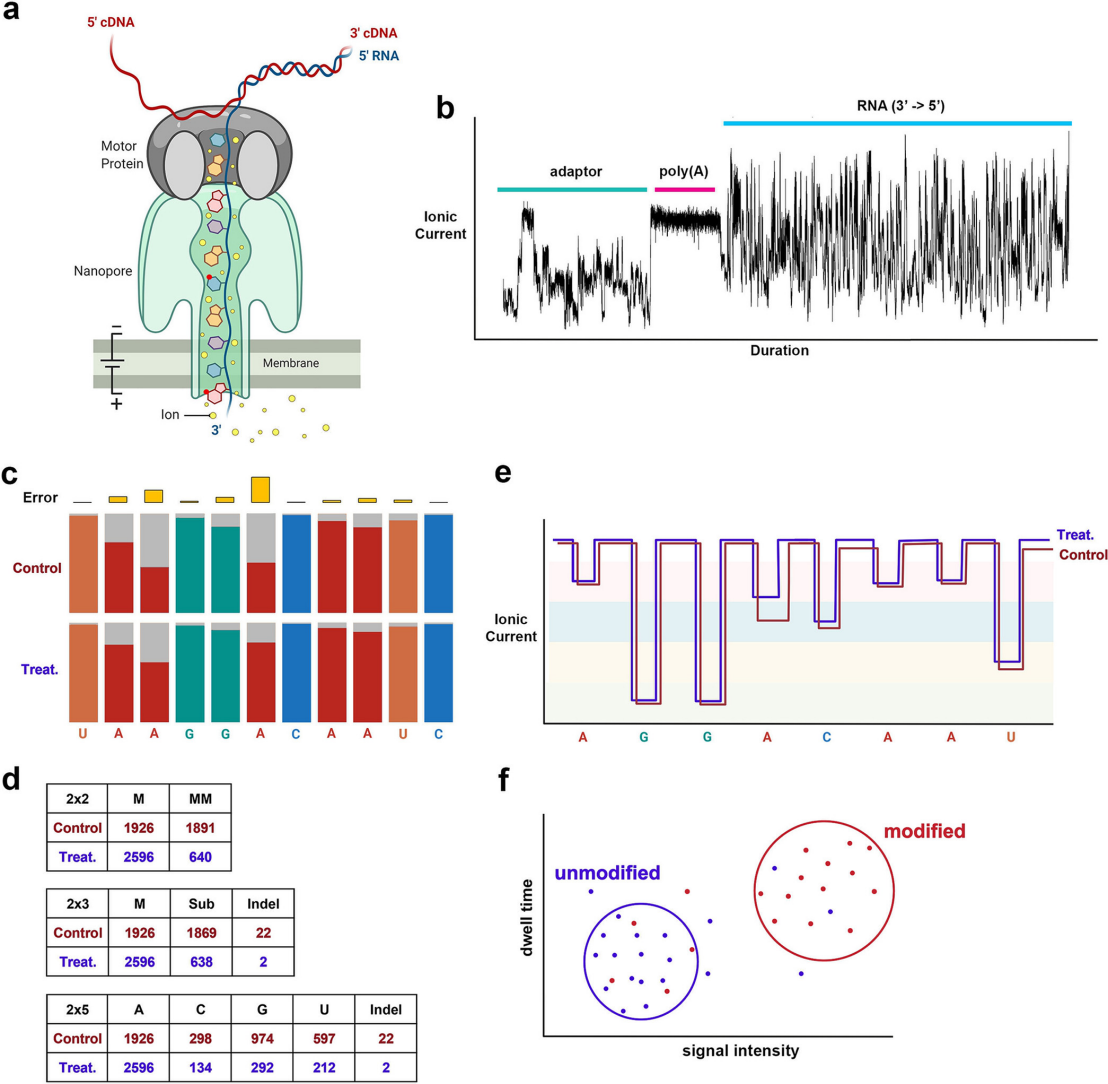
Overview of the nanopore direct RNA sequencing approach and RNA modification detection strategies. (a and b) DRS libraries comprise RNA-cDNA hybrids that are unwound by a helicase when docked to a membrane-embedded nanopore. (a) The RNA strand is selectively pushed through the nanopore in a 3′-to-5′ direction, disturbing the general flow of ions through the pore. (b) Ammeters placed within the narrowest part of the pore measure changes in ionic flow (current), which can subsequently be partitioned into sections attributed to the DNA adapter, poly(A) tail, and body of the sequenced RNA, here visualized as a squiggle plot (124). (c and d) Putatively modified ribonucleotides are identified through either error rate or signal-level analyses. (c) Error rate analyses identify individual positions within RNAs that show significantly different error profiles between two conditions (e.g., control versus treatment). Here, the proportion of reads containing the correct nucleotide at a given position is shown in colors other than gray, while the proportion of reads containing erroneous bases at the same position is shown in gray. The difference in calculated error rate is shown in gold. (d) Error profiles undergo statistical analyses in the form of 2 × 2, 2 × 3, or 2 × 5 contingency tables that are subsequently corrected for multiple testing. M, match; MM, mismatch. (e) For signal-level analyses, the initial process of resquiggling assigns ionic flow features (signal intensity, dwell time, etc.) to their corresponding nucleotides within individual reads. (f) These features either undergo a comparative analysis (between data sets) or are evaluated using data from a prebuilt model generated by machine and/or deep learning.

**FIG 2:**
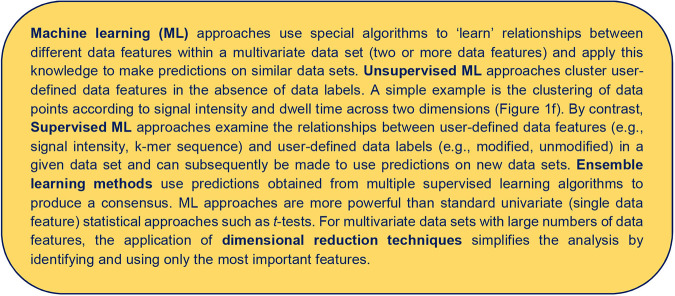
Brief overview of machine learning.

In 2017, Oxford Nanopore Technologies (ONT) described a first approach to sequencing the full length of native RNA molecules extracted from a cellular context (77). They termed this technique direct RNA sequencing (dRNA-Seq or DRS). The construction of DRS libraries remains remarkably quick and simple at ~2 h. The standard approach utilizes 250 to 500 ng of isolated poly(A) RNA as input for a ligation reaction in which a double-stranded DNA adaptor is ligated to the 3′ end of the poly(A) tail (77). Variations to this approach include the use of custom adaptors that allow targeting of specific nonpolyadenylated RNAs (78) or the use of poly(A) and rRNA depletion prior to artificial polyadenylation and capture, e.g., to capture nascent transcription (79). Following ligation, a reverse transcription step produces an RNA-cDNA duplex that stabilizes the RNA strand and reduces secondary structure formation before a second ligation allows the attachment of an engineered motor protein that allows nanopore docking and unwinding of the RNA-cDNA duplex. The DRS library is subsequently loaded onto a flow cell for sequencing. The flow cell contains an electrically resistant polymer membrane separating two electrodes, across which can be applied an electrical current. A series of protein nanopores are embedded within the polymer, each with its own sensor chip that continuously records the flow of ions (ionic current) passing through the nanopore. Upon docking with a nanopore, the motor protein unwinds the RNA-cDNA duplex, ratcheting the RNA molecule through the nanopore in the 3′-to-5′ direction at a constant rate (~70 nt/s) (Fig. 1a). The flow of ionic current is measured within the narrowest part of the nanopore by an ammeter with contact points set a distance apart that roughly corresponds to 5 nucleotides (Fig. 1b). Thus, at any given time, the ionic current recorded generally represents a function of 5 nucleotides (5-mer). The composition of nucleotides in this 5-mer is subsequently derived by “base calling.” Here, ionic current changes are segmented as discrete “events” in which the duration (dwell time), amplitude (signal intensity), and variance are recorded. Machine-learning (ML) (Fig. 2) algorithms (for an excellent review of ML geared toward biologists, see reference 80) subsequently convert “events” into “base calls,” with each identified nucleotide being assigned a probability score that denotes the predicted accuracy of the call. While several base calling algorithms exist, the most commonly used is Guppy. Developed by ONT, Guppy uses a recurrent neural network (a form of deep learning [81]) trained on synthetic data sets comprising 5-mers of all possible A, C, G, and T or U combinations to predict the underlying nucleotide sequences with variable accuracy: 94 to 98% for DNA and 86 to 91% for RNA (82–84). At the DNA level of Guppy base calling, the training data sets have recently undergone further expansion to enable the native detection of specific DNA modifications (6mA and 5mC). However, such support is not yet available for RNA modifications. This is due in part to the vast numbers and diversity of RNA modifications (>170) that exist. Consider, for example, a 5-mer of unmodified ribonucleotides (A, C, G, U). Here, there are 4^5^ (1,024) potential sequence combinations that must be considered during base calling. Add in just a few modified ribonucleotides such as m^1^A, m^6^A, and pseudouridine, and the number of sequence combinations changes to 7^5^ (16,807). There thus remains a burden on finding alternative approaches to detecting RNA modifications in DRS data sets, a challenge that has been embraced by the wider scientific community. At the time of writing, nanopore DRS approaches had been used to detect a wide range of RNA modifications, including m^6^A, internal m^7^G, m^5^C, Ψ, Nm, and 5-hydroxymethylcytosine (5hmC) (85). This was made possible by distinct analytical approaches that we broadly classify as “error rate” and “signal-level” methodologies (Fig. 1c to f; Table 1).

**TABLE 1:** Nanopore-based RNA modification detection softwares and their characteristics

Analysis type and tool	Modification(s) reported^*a*^	Modifications targeted^*b*^	Approach	Stoichiometry estimates	Reference
Error rate					
DRUMMER	m^6^A	Any	Comparative	No	54
Eligos2	m^6^A	Any	Individual/comparative	No	88
JACUSA2	m^6^A	Any	Comparative	No	90
DiffErr	m^6^A	Any	Comparative	No	89
EpiNano	m^6^A, Ψ	Any	Individual/comparative	No	86
					
Signal level					
Tombo	m^6^A/m5C	Any	Individual/comparative	No	92
MINES	m^6^A	m^6^A only	Individual	No	100
nanom6A	m^6^A	m^6^A only	Individual	No	101
nanoRMS	Ψ, Nm	Any	Comparative	Yes	98
Nanocompore	m^6^A, Ψ	Any	Comparative	No	96
m6Anet	m^6^A	m^6^A only	Individual	Yes	102
xPore	m^6^A	Any	Individual/comparative	Yes	97
Penguin	Ψ	Ψ only	Individual	No	105
Yanocomp	m^6^A	Any	Comparative	Yes^*c*^	99
NanoPsu	Ψ	Ψ only	Individual	Yes	106
DENA	m^6^A	m^6^A only	Individual	No	104

a m^6^A, *N*^6^-methyladenosine; m^5^C, 5-methylcytosine; Ψ, pseudouridine; Nm, 2′-*O*-methylation.

b Any, theoretically capable of detecting any RNA modification that alters ionic current.

c Stoichiometry estimates claimed but not evidenced at time of writing.

## INFERRING RNA MODIFICATIONS THROUGH BASE CALL ERROR RATES

The presence of modified ribonucleotides within an RNA has a marked effect on the ionic flow passing through a nanopore, a feature that directly affects the accuracy of downstream base calling. This generally manifests as an increased probability of one or more wrong nucleotides being assigned to a given 5-mer. By calculating the error rate (% incorrect base calls) at each position across a set of identical reads, one can identify hot spots of increased error rate. However, hot spots may also originate from natural within-sample polymorphisms and the presence of homopolymers, as well as stochastic factors. To correct for this, most error rate approaches utilize a comparative strategy in which hot spots are considered real only if they appear in a data set known to contain a given modification (e.g., m^6^A) compared to an identical data set in which the modification is absent (Fig. 1c). This latter strategy is achieved in many ways, including inhibition, silencing, or knockout of specific proteins, or else through the generation of *in vitro*-transcribed (IVT) RNA. Putative modified ribonucleotides are subsequently identified through one or more statistical tests, usually based on contingency tables (Fig. 1d), and the inclusion of biological replicates. At the time of writing, five softwares applying the error rate approached had been released (Table 1).

EpiNano was the first software to make use of the error rate approach by relying on a supervised learning approach (in this case, a support vector machine algorithm trained on synthetic m^6^A-modified and unmodified *in vitro*-transcribed constructs) to predict m^6^A modifications at RRACH motifs within yeast strains (86). This has since been replaced by a comparative approach termed EpiNano-Error, which uses features such as base quality score, the presence of indels, and/or mismatches to identify differences in error patterns between two matched samples (i.e., control versus treatment) and was recently used to detect m^6^A within circular RNAs (circRNAs) (87). Another tool, Eligos2, predicts modified sites by considering the difference in error rate (between two conditions) at a given position as well as at the two upstream and downstream flanking positions. Each position is tested using a Fisher exact test on a 2 × 2 contingency table containing matches and mismatches (substitutions, insertions, and deletions) (Fig. 1d). *P* values from each test are corrected via the Benjamini-Hochberg method and an odds ratio test used to identify putatively modified positions. Synthetically modified IVT RNA was initially used to make predictions on nine different RNA modifications (m^6^A, m^1^A, ψ, m^7^G, inosine, hm^5^C, m^5^C, 5-methoxyuridine [5moU], and 5-formylcytosine [f^5^C]), with resulting area under the receiver operating characteristic (ROC) curves (AUCs) ranging from ~0.54 to 0.92 (i.e., poor to great). Eligos2 was subsequently used to demonstrate that the sequence contexts of putative m^6^A sites identified in yeast and human data sets were enriched for DRACH motifs (88). DRUMMER parses each position within a comparative alignment into a 2 × 5 contingency table (Fig. 1d) that is evaluated using a G test and an odds ratio test. The resulting *P* values are corrected for multiple testing, and a list of candidate sites are returned. Further filtering of data can be performed by estimating and correcting for background noise. An early version of DRUMMER was used to identify m^6^A within adenovirus type 5 and mouse transcriptome data sets (54). Conceptually similar to DRUMMER, DiffErr also parses comparative alignments into a series of 2 × 5 contingency tables, one per position in the alignment. A G test is applied and, where *P* is <0.05, a G test for homogeneity between replicates of the same condition is performed. Positions at which the G test score between replicates is higher than the G test score between conditions are excluded, and the remaining positions undergo multiple testing correction. Sites with an adjusted *P* value of <0.05 and a log fold change (logFC) of >1 in control versus treatment samples are classified as methylated. The authors applied DiffErr to study m^6^A in Arabidopsis thaliana with 66% of predicted sites being within five bases of a miCLIP peak (89).

While each of the above tools can theoretically detect any RNA modification that produces current fluctuations during nanopore sequencing, JACUSA2 is presently limited to m^6^A detection. JACUSA2 considers three potential events that may arise from errors (substitutions, insertions, deletions) and examines only 5-mers with a central A (e.g., NNANN). This results in a 30-feature matrix (3 events, 5 k-mers, 2 conditions) that subsequently undergoes dimensionality reduction to identify characteristic patterns of m^6^A modifications. Using a comparative approach, the authors identified multiple m^6^A sites located in DRACH sequence contexts, and when this was extended to focus on the EEF2 gene locus, they reported both a novel and previously identified m^6^A site (90).

## INFERRING RNA MODIFICATIONS THROUGH CHANGES IN SIGNAL INTENSITY

Signal-level approaches detect modified ribonucleotides by directly leveraging the raw electrical signal information collected during nanopore sequencing runs. As a first step, every signal-level approach requires “resquiggling” of the data. Resquiggling describes the process by which the raw signal for each individual read is dissected into “events” that are overlaid onto read alignments by using specialist algorithms included in the nanopolish (91) and Tombo (92) packages (Fig. 1e). The resulting signal information is used to either (i) train a model or (ii) perform comparative analyses between two experimental conditions (Table 1). Training a model uses the k-mer information and signal intensities as features and requires a list of known modification statuses to make a prediction. Software packages invoking this method thus have the benefit of running without a control sample, if the experimentally desired model already exists. However, such training models require input data that can be obtained only through orthologous data sets (e.g., miCLIP) or manual curation from online databases (e.g., REPIC [93]). By contrast, the comparative approach compares signal data from two distinct data sets (e.g., treatment versus control) to identify sites at which signal information deviates. Signal-level analyses are considered to have far greater potential than error rate approaches, and this has resulted in a wider range of tools being developed, again producing a divide between (i) those that can theoretically detect any RNA modification that changes the ionic flux and (ii) those that are specific for individual modifications (e.g., m^6^A).

Starting with the former category, Tombo supports the detection of both DNA and RNA modifications (92). Three specific methods are available depending on the available data sets: alternative, *de novo*, and sample comparison. For DRS data sets, the sample comparison approach is specifically recommended and provides two models for analysis (i) model_sample_compare, and (ii) level_sample_compare. These methods compare two sets of reads to identify differences at each reference position based on signal-level distributions by using standard univariate statistical tests, i.e., the KS test, the U test, and the *t* test. While the original presentation of this tool focused on DNA modifications, Tombo has recently been used to look at m^5^C modifications in the transcriptomes of porcine reproductive and respiratory syndrome virus (94) and Arabidopsis thaliana (95). Nanocompore uses a comparative approach to make RNA modification calls by initially grouping reads belonging to each reference transcript and then assigning their corresponding median signal intensity and dwell time to each transcript position (96). The two conditions are compared on a positional basis using either a univariate pairwise test or a bivariate classification method based on a two-component (dwell time versus signal intensity) Gaussian mixture model (GMM), a form of supervised learning. GMM clustering is followed by a logistical regression test which determines the significance in difference of reads between conditions. Finally, *P* values are corrected for multiple testing. Synthetically modified oligonucleotides were used to benchmark the accuracy of Nanocompore for numerous different modifications (including m^6^A, inosine, ψ, m^5^C, and m^1^G) before analysis of m^6^A in yeast and human RNAs. Conceptually similar to Nanocompore, xPore also uses a GMM to compare differences in signal intensity (but not dwell time) between modified and unmodified data sets (97). However, it also adds in prior information of a theoretical signal distribution of unmodified RNA to further guide the Gaussian model. Using this model, signal properties such as the mean and variance are used to estimate the fraction of modified reads and assign a modification probability. A two-step intermediate filtering step is included to reduce false positives, with the remaining sites undergoing a Z-test on differentially modified rates between samples. In their analysis of m^6^A modified sites, the authors focused on positions with a central adenine (NNANN), using DRS from wild-type (WT) and METTL3 knockout (KO) HEK293 T cells, and they obtained an AUC of 0.86. However, when the analysis was extended to all k-mers, the rate of false positives increased. nanoRMS utilizes signal intensity, dwell time, and base quality/probability information to perform either unsupervised or supervised clustering that assigns reads into modified and unmodified groups. When running a supervised clustering method, which is generally suited to low-stoichiometry contexts such as m^6^A on mRNA, comparative data sets (e.g., treatment versus control) are required. By contrast, unsupervised clustering is used to predict RNA modifications in a single sample alone. The limitation is that this is generally able to predict only specific RNA modifications in high-stoichiometry contexts, e.g., pseudouridine in rRNAs. In the original publication, nanoRMS processed comparative DRS data sets to predict pseudouridine modifications within yeast RNAs and, further, identified two new pseudouridines in yeast 15s mitochondrial rRNA (98). Finally, Yanocomp uses a comparative analysis approach to identify modifications by using a 5-mer sliding window to fit a multivariate GMM to separate data into modified and unmodified distributions (99). Yanocomp has been successfully applied to the identification of m^6^A modifications within the *A. thaliana* transcriptome, which showed a significant overlap with sites identified by the orthologous miCLIP method.

In the category of modification-specific tools, four are designed specifically for the detection of m^6^A while two are designed to identify pseudouridine. The common feature of modification-specific tools is that they use machine learning approaches to identify putatively modified sites. This strategy obviates the need for a comparative analysis (e.g., treatment versus control) and may be particularly useful for screening data sets and generating preliminary analyses. The first of these, MINES, specifically detects m^6^A modifications by harnessing Tombo’s *de novo* algorithm by filtering putatively modified sites to keep only those located within a 30-nucleotide window centered within a DRACH motif (100). This approach reduces computational load but restricts the analysis to m^6^A modifications and indeed will not detect m^6^A installed outside of DRACH motifs (e.g., DRACG). The stoichiometric values obtained postfiltering were combined with publicly available miCLIP results to train a classifier. The prediction accuracy of the classifier ranged from 67 to 83% and was heavily reliant on DRACH sequence contexts. Nanom6A extracts features such as the median, mean, and standard deviation of signal for each 5-mer and uses these to train an ensemble learning model on RRACH motifs (101). To validate their model, they used Nanom6A to predict modified and unmodified reads within a synthesized mRNA data set with known m^6^A and non-m^6^A ratios. Validation was extended in HEK293 cells by quantifying the loss of m^6^A following METTL3 silencing. In addition, they used Nanom6A across different biological systems with known locations of m^6^As and compared their results against those of different biological/computational approaches. Here, 81% of m^6^A-modified mRNAs predicted by Nanom6A were validated by meRIP-Seq. At single-base resolution, 49% of m^6^A predictions correlated with meRIP-Seq peaks. m6Anet uses a combination of signal- and sequence-level features of DRS data sets to predict a probability of modification at each individual site using a multiple instance learning framework (102). To train their model, the authors used the HCT116 cell line with matched m^6^A-cross-linking-exonuclease sequencing (m^6^ACE-Seq) (103)-obtained modification labels. This approach yielded an AUC of 0.83 across DRACH motifs in two different human cell lines. DENA uses signal features (mean, median, standard deviation, dwell time, and base quality) obtained from Tombo resquiggling to train a neural network to make m^6^A modification calls (104). Training data were obtained by analysis of *A. thaliana* DRS data sets, in which DiffErr (99) was initially used to identify sites at RRACH motifs that contained a differential “error” within 5 nucleotides between the WT and KO. Electrical signals at these sites from either WT or KO reads were used as training labels, m^6^A or A, respectively. For the 12 possible RRACH motifs, the AUC ranged from 0.90 to 0.97 on the test data set, along with 90% accuracy by comparing these sites to m^6^A sites detected by miCLIP.

Penguin focuses on detection of pseudouridine and extracts 5-mers, mean, standard deviation, and length of signal. 5-mers are further filtered to retain only those containing a uracil at the center (105). A gold standard set of modified bases contained within HEK293 and HeLa cell lines were used as labels and were merged with the output from nanopolish. Three unsupervised models were trained and used to benchmark the two cell lines, achieving accuracies between 85 and 93%. Similar results were achieved when training was done specifically on HEK293 T cells and tested on an independent cell line (i.e., HeLa). NanoPsu uses 12 different base calling error features to identify pseudouridine by using supervised learning (106). To train the model, rRNAs isolated from human, yeast, Caenorhabditis elegans, Drosophila melanogaster, and human fecal bacteria were split into two subsets. For each sample, half underwent Illumina bisulfite sequencing to map pseudouridine sites (2,142 sites) which were used as training labels, while the other half underwent nanopore DRS sequencing to obtain training features. After filtering for pseudouridine sites that passed a read coverage of 20, 640 modified and 689 randomly selected unmodified U sites were used for training. This resulted in an AUC of 0.938 on the test data set, with downstream analysis on HeLa cells (in the presence/absence of interferon treatment) further validating their results.

## (CURRENT) LIMITS IN THE DETECTION OF MODIFIED RIBONUCLEOTIDES BY NANOPORE SEQUENCING

Taken together, it is clear that a wealth of methodologies exist by which DRS data sets can be used to identify putatively modified nucleotides. The choice of error rate versus signal-level approaches offers researchers semiorthologous approaches with which to analyze the same data set. However, caution must be applied here, as a recent study demonstrated that both error rate and signal-level approaches have “blind spots” that may result in potential false negatives (i.e., the presence of a modified nucleotide reported by error rate but not signal-level approaches and vice versa) (98). Moreover, several of the above-mentioned tools (xPore, m6Anet, nanoRMS, NanoPsu) offer stoichiometry estimates at the level of individual isoforms, although the accuracy of these estimates remains questionable. In part, this is due to the lack of an available “ground truth” data set in which the location and abundance of all RNA modifications is known. Given the technical challenges and excessive cost associated with generating such a data set, it seems unlikely that this issue will be rectified soon. When considering which methodology/tool to use, it is worth noting that while powerful and generally easier to implement, error rate methodologies are limited to the level of RNA isoforms rather than individual RNAs and their power is greatest where read depths are very high, as sensitivity generally scales with depth of sequencing (107). Signal-level methodologies are, at present, also limited to the level of RNA isoforms but require significantly lower read depths to make accurate predictions and retain the promise of allowing analysis of individual reads in the future. While none of these methods yet achieves the ultimate goal of identifying the presence and position of modified ribonucleotides within individual RNAs, their potential remains far greater than short-read sequencing approaches. Indeed, the primary difference remains that the experimental sides of short-read approaches (meRIP-Seq, miCLIP-Seq, etc.) are long and complex while the subsequent computational analysis of sequencing data is generally simple due to the existence of many well-established and robust pipelines. By contrast, DRS data sets can be generated in just a few hours with little technical expertise required. However, computational analyses are currently more complex due to a relative lack of robust, well-validated pipelines.

## NANOPORE-MEDIATED DETECTION OF RNA MODIFICATIONS IN VIRAL CONTEXTS

At the time of writing, just six of the above-described tools had been applied to the analysis of viral DRS data sets: DRUMMER, Nanocompore, Eligos2, Tombo, MINES, and m6Anet. The first reported use of DRS to examine RNA modifications in viral contexts was published in 2019 by Viehweger and colleagues (108). Here, several subgenomic RNAs (sgRNAs) of the human alphacoronavirus HCoV-229E were reported by Tombo to contain 5-methylcytosine (m^5^C) in both the conserved leader sequence (LS) and downstream open reading frames (ORFs). The validity of the reported signal has not, at the time of this writing, been confirmed by orthologous approaches (e.g., bisulfite sequencing). The subsequent emergence of the betacoronavirus SARS-CoV-2 in late 2019 led to a rash of studies aiming to identify and characterize the role of RNA modification in coronavirus biology (109–114). First among these was a delineation of the SARS-CoV-2 transcriptome architecture that included a description of 41 locations containing putatively modified ribonucleotides (109). These sites were identified by Tombo through a comparative analysis of native SARS-CoV-2 sgRNAs with 15 partially overlapping IVT fragments that spanned the entire SARS-CoV-2 gRNA. Sequence analysis identified the most common motif at these locations to be AAGAA-like, and these were particularly enriched at the 3′ end of viral sgRNAs and more commonly on longer viral transcripts (gRNA, S, 3a, E, and M) than shorter ones (6, 7a, 7b, 8, and N). Of note, the authors also performed a comparative m^5^C analysis and observed numerous signal-level changes using pretrained models. However, these changes were also observed in the unmodified (IVT) controls, suggesting that these are false positives. A second study soon followed in which a small number of sgRNAs from SARS-CoV-2 and the related human betacoronavirus HCoV-OC43 were shown to harbor m^6^A modifications. The first of these utilized DRUMMER and Eligos2 to perform comparative analyses of DRS data sets derived from SARS-CoV-2 (A549^+ACE2^) and HCoV-OC43 (MRC-5)-infected cells that were treated with either a specific METTL3 inhibitor or a noninhibiting control (110, 115). These data were validated using the orthogonal meRIP-Seq methodology (61, 110) and also served to show that inhibiting the enzymatic activity of METTL3 led to reduced titers of both SARS-CoV-2 and HCoV-OC43. The installation of m^6^A on a small number of SARS-CoV-2 sgRNAs was further validated by additional studies (111, 112) using comparative DRS approaches allied with Tombo, MINES, and m6ANet. Another recent study utilized Tombo to show that the SARS-CoV-2 epitranscriptome remained stable during infection of Vero cells (113). While the identity of RNA modifications at the individual modified sites was not reported, the authors noted that at least two sites appeared unique to the gRNA and were not found on sgRNAs. The validity of these findings remains uncertain, however, due to a lack of orthologous confirmations. Finally, the presence of five pseudouridine modifications in the SARS-CoV-2 transcriptome was revealed by comparative analysis of SARS-CoV-2 DRS data sets and synthetic RNAs, two of which were confirmed through *in vitro* assays (114).

Beyond human coronaviruses, the use of nanopore DRS in detecting viral RNA modifications has thus far been limited, with just two studies published to date (54, 116). The first examined the role of m^6^A in the life cycle of adenovirus serotype 5. Here, DRS data sets were derived from adenovirus-infected A549 cells in which METTL3 was present or knocked out (54). A comparative analysis of these data sets, which included two biological replicates per condition, was performed using alpha versions of DRUMMER and Nanocompore and at both the exome and isoform levels. Both tools identified a large number of putatively modified sites in both early and late viral transcripts, with the majority (~80%) reported by both tools and located within DRACH motifs. Further analysis of the DRS data sets generated in the absence of METTL3 showed a significant reduction in the splicing efficiency of late viral RNAs. The second study examined the role of m^6^A in the life cycle of herpes simplex virus 1 (HSV-1) (116). Remarkably, HSV-1 induces relocalization of multiple host proteins associated with RNA modifications from the nucleus to the cytoplasm and, with this, a loss of RNA modifications, including m^6^A, from both host and viral mRNAs in a manner mediated by the viral ICP27 protein. The losses from human RNAs were determined by a comparative analysis of DRS data sets derived from HSV-1-infected fibroblasts and IVT RNAs representing the human gamma actin (ACTG1), while the loss of modified ribonucleotides from the viral RNAs was determined by a comparative analysis of infections of normal human dermal fibroblasts (NHDFs) with an HSV-1 wild type or an ICP27 null mutant. The comparative analysis was performed using an alpha version of DRUMMER, which reported that only around 25% of modified sites were located within or proximal to AC dinucleotides within DRACH motifs. It was thus inferred, but not validated, that the remaining sites represent other modified ribonucleotides.

## EXPERIMENTAL CONSIDERATIONS WHEN USING NANOPORE SEQUENCING TO IDENTIFY RNA MODIFICATIONS

As is demonstrated above, the breadth and depth of computational approaches for detecting modified ribonucleotides in nanopore DRS data present virologists with a wide range of analytical options but also require a careful consideration of the underlying aims of individual experiments and the resulting data quality. Based on our own experiences with adenoviruses, herpesviruses, and betacoronaviruses, we have curated a list of the following parameters that we consider to be critical for consideration in the design and execution of nanopore-based RNA modification studies in viral contexts.

### (i) Annotation quality.

RNA modification detection tools require that DRS data sets are aligned against the transcriptome of the organism of interest. In viral contexts, this remains problematic, as very few viruses possess high-quality annotations. In the absence of such annotations, alignment of DRS reads often produces a subset of artefactual results in which the annotation for a given sequence read does not exist and leads to a subset of reads being assigned to an alternative transcript isoform instead. This typically hinders RNA modification analysis through the generation of false-positive and/or false-negative results. This can be remedied by either (i) generating a new high-quality transcriptome annotation or (ii) limiting the analysis to the exome level (i.e., aligning DRS data to the genome rather than the transcriptome). The former remains a complex, time-consuming, but ultimately valuable undertaking that can be partially accomplished using computational annotation tools such as StringTie2 (117) and FLAIR (118). The latter approach is simple and quick and can be accomplished with tools such as DRUMMER, Eligos, and Yanocomp, albeit at the expense of sensitivity and specificity (54).

### (ii) Prefiltering of aligned read data.

Regardless of whether pursuing exome- or isoform-level alignments, filtering of DRS read alignments prior to RNA modification detection can significantly improve sensitivity and accuracy. Standard measures should include screening for and removing adapter sequences from DRS reads, as well as removing secondary and supplementary alignments. This latter measure may also include rejecting individual reads with multiple primary alignments (i.e., they cannot be uniquely assigned to a single transcript isoform). A further step that is particularly relevant for isoform-level (i.e., transcriptome) analyses involves removing all primary read alignments that do not extend to within 50 nt of the defined 5′ end of a given transcript.

### (iii) Biological replicates.

The use of at least two biological replicates per condition is recommended. Several tools have inbuilt support for such analyses (DRUMMER, Eligos, Nanocompore, xPore, EpiNano), while others require additional scripting to merge results from individual analyses. The principal benefit of including biological replicates is to enhance the identification of consistently modified sites while also providing measures of stochastic variation.

### (iv) Manipulating RNA modification abundance.

Comparative analyses rely on comparing conditions in which a given modified ribonucleotide is present at differing levels. In our experience with m^6^A, inhibition of METTL3 enzymatic activity or the generation of METTL3 conditional knockout cell lines significantly enhances sensitivity relative to RNA silencing strategies. Error rate analysis tools are particular insensitive to RNA silencing strategies, presumably due to reduced stoichiometric differences between conditions. The use of IVT RNAs is another increasingly popular strategy, although this generally limits comparative approaches to reporting the presence/or absence, but not the identity, of all modifications within the RNA isoform being compared.

### (v) The right tool for the right job.

Choosing the right tool for a given analysis is a complex process. Read depth is a critical factor for several tools (DRUMMER, Eligos) (88, 107). Others favor specificity over sensitivity (EpiNano, m6Anet, MINES). Some (Nanocompore, xPore, m6Anet) even allow for estimates of stoichiometry, although these require careful orthogonal validation. Where possible, we recommend performing analyses with at least two or more distinct nanopore RNA modification tools and comparing outputs. This can help identify both false positives and false negatives that may arise from the intrinsic biases of a given tool. Due to the ease of installation, comprehensive documentation, and flexibility regarding how transcriptome alignments can be preprocessed, we have had particular success in viral contexts with DRUMMER, Nanocompore, and nanoRMS, although a comprehensive formal comparison of all tools has not been undertaken.

## OUTLOOK

The field of nanopore sequencing is moving rapidly, pushed by constant biological and computational innovations. As noted above, native support for the identification of several DNA modifications was recently integrated into Guppy, and it is reasonable to expect similar support for some RNA modifications (e.g., m^6^A) in the coming years. This is driven by ongoing improvements in the DRS chemistry that will further reduce error rates and make modified ribonucleotides easier to distinguish from their unmodified counterparts. Whether such improvements will ultimately limit the utility of error rate approaches to RNA modification detection remains uncertain. We further anticipate that computational approaches for RNA modification detection will increasingly focus on the accurate measurement of stoichiometry, through both refinement of existing tools such as Nanocompore, xPore, and nanoRMS and the development and release of novel tools.

In looking further toward the future, we expect that comparative analysis approaches for profiling RNA modifications will eventually become defunct, replaced by a combination of native detection during base calling and the use of advanced signal-level algorithms capable of reporting multiple different types of RNA modifications within a single analysis (e.g., m^6^A, m^1^A, pseudouridine, etc.). Such developments would also place within reach the ultimate goal of identifying the presence, identity, and specific location of modified ribonucleotides within individual RNA molecules.

To conclude in the context of viral RNA biology, the key limitation at present remains the general lack of well-annotated transcriptomes. Without these, viral RNA modification studies will be restricted to exome-level analyses that lack resolution. While resolving this will take time, it is notable that an increasing number of studies are addressing this issue across the viral spectrum (109, 119–123). Where high-quality transcriptome annotations already exist, it is clear that RNA modification detection using nanopore sequencing has provided significant advances that would simply not be possible using other approaches. This will only be enhanced further in the future.
